# Comparative analysis of PD-L1 expression and molecular alterations in primary versus metastatic lung adenocarcinoma: a real-world study in China

**DOI:** 10.3389/fonc.2024.1393686

**Published:** 2024-09-11

**Authors:** Gang Chen, Yang Yu, Youchao Qi, Guangxu Li, Ning Li, Fande Meng, Wujie Wang, Rong Shen

**Affiliations:** ^1^ Department of Thoracic Surgery, Shandong Provincial Hospital Affiliated to Shandong First Medical University, Jinan, Shandong, China; ^2^ Department of Thoracic Surgery, The Second People’s Hospital of Dezhou City, Dezhou, Shandong, China; ^3^ Department of Radiotherapy, The Second People’s Hospital of Dezhou City, Dezhou, Shandong, China; ^4^ Department of Internal Medicine, Changle County Traditional Chinese Medicine (TMC) Hospital, Weifang, Shandong, China; ^5^ Department of Interventional Medicine, The Second Hospital, Cheeloo College of Medicine, Institute of Tumor Intervention, Shandong University, Jinan, Shandong, China; ^6^ Department of Minimally Invasive Oncology, Shandong Provincial Hospital Affiliated to Shandong First Medical University, Jinan, Shandong, China

**Keywords:** lung adenocarcinoma, gene mutation, PD-L1, metastatic lesion, CDKN2A

## Abstract

**Objectives:**

Programmed death-ligand 1 (PD-L1) is the only Food and Drug Administration-approved biomarker for monitoring response to immune checkpoint inhibitor (ICI) therapy in patients with lung adenocarcinoma. Understanding the nuances of molecular phenotypes, clinical attributes, and PD-L1 expression levels in primary and metastatic lung adenocarcinoma may help predict response to therapy and assist in the clinical management of lung adenocarcinoma.

**Methods:**

A total of 235 primary and metastatic lesion specimens from patients with non-small cell lung cancer (NSCLC) an institution in Shandong, China were analyzed. PD-L1 expression was assessed by immunohistochemistry using the 22C3 antibody, and the molecular phenotype was determined by next-generation sequencing of 450 genes. The molecular phenotypes of the primary and metastatic lesions were compared.

**Results:**

Elevated PD-L1 expression was significantly associated with advanced and metastatic disease (P = 0.001). The distribution of PD-L1 expression varied based on the anatomical location, showing a higher frequency of elevated PD-L1 expression in distal metastases than in the primary tumor. Metastatic lesions exhibited a higher proportion of carcinogenic pathway gene alterations and a greater number of DNA damage-repair pathway gene alterations than the primary lesions. Notably, *CDKN2A* copy number deletions were more prevalent in metastatic lesions than in primary lesions. Clinical data stemming from research conducted at the Memorial Sloan Kettering Cancer Center revealed an association between the absence of CDKN2A expression and a poorer prognosis in stage I lung adenocarcinoma.

**Conclusion:**

Samples of metastatic tumors exhibited a higher proportion of elevated PD-L1 expression, a greater number of pathway alterations, and a higher occurrence of *CDKN2A* copy number deletions than primary samples. This highlights the importance of reinforcing the clinical management and follow-up of patients with *CDKN2A* deficiency, particularly within the subset of stage I lung adenocarcinoma.

## 1 Introduction

China accounts for one-fifth of the global population. Lung cancer was ranked as the fourth leading cause of death in China between 1990 and 2017, with more than 20% increase in the lung cancer death rate during this period (1). Shandong Province, representing approximately 14% of China’s population with approximately 100 million inhabitants, exhibited an improved 5-year survival rate among patients with lung cancer based on cancer registration data from 2012 to 2018 (2). This progress can be partially attributed to widespread gene identification efforts and active engagement in targeted therapy and immunotherapy. Notably, the 5-year lung cancer survival rate in Shandong Province was only 24.4% during this period, indicating the need for ongoing efforts to improve lung cancer survival (2).

The advancement of large-scale gene sequencing technology has led to an increasing availability of molecular-targeted therapies for lung cancer. This technology, along with extensive array detection, has significantly enhanced the understanding of the mechanisms underlying lung cancer metastasis and recurrence. Previous studies have shown a correlation between the metastatic load of lung cancer and chromosomal instability (3), particularly increased copy number instability in patients with brain metastases from lung cancer (4). Moreover, studies have shown higher expression levels of programmed death-ligand 1 (PD-L1), an immunotherapy marker for lung cancer, in distant metastatic lesions than in primary lung lesions (5). These findings prompted us to investigate the differences between primary and metastatic lung cancer lesions.

This study aimed to perform a comparative analysis of DNA-level mutations and PD-L1 expression levels in primary and metastatic lesions derived from a single-center lung cancer cohort in Shandong, China. Additionally, this study aimed to identify key genes associated with survival to advance the clinical management of lung cancer.

## 2 Materials and methods

### 2.1 Patients

Following approval from the Ethics Committee of Shandong Provincial Hospital, targeted sequencing and PD-L1 immunohistochemistry (IHC) testing were conducted on surgical tissue samples obtained from patients diagnosed with lung adenocarcinoma. The requirement for informed consent was waived because the study was a retrospective study that used leftover surgical specimens.

These tests were performed (6). Tumor tissue samples obtained between April 2018 and February 2022 were selected for analysis, and the medical and pathological records of patients were thoroughly reviewed and the relevant data were extracted.

### 2.2 Targeted sequencing

Targeted sequencing was performed using to analyze all encoded exons encompassing 450 genes, along with the TERT promoter and introns of 39 genes, as described previously (6). The somatic changes identified included mutations and copy number alterations. Specifically, MuTect, Pindel, and EXCAVATOR were used to determine single nucleotide variations (SNVs), insertions and deletions (InDels), and copy number variations (CNVs), respectively. In addition, internally developed algorithms were used to screen for gene rearrangements. To ensure accuracy, all identified variations underwent manual examination using the Integrative Genomics Viewer to minimize potential errors.

### 2.3 PD-L1 test

PD-L1 expression scores were determined by thoracic pathologists and reported as the percentage of tumor cells exhibiting membranous staining. In this study, PD-L1 subgroups were categorized as negative (PD-L1 tumor proportion score [TPS] < 1%), moderate (1% ≤ PD-L1 TPS < 49%), and high (PD-L1 TPS ≥ 50%). PD-L1 immunohistochemistry (IHC) was performed using 28-2 or 22C3 antibodies.

### 2.4 Gene and pathway analysis

The analysis focused on assessing the distribution and enrichment of individual genes within distinct subgroups by investigating previously identified carcinogenic or potentially carcinogenic mutations. A gene pathway list was developed by cross-referencing gene lists from the literature (5) with the overlapping genes identified through the meta-tracking products.

### 2.5 Statistics

Comparisons between proportions were performed using Fisher’s exact test or Pearson’s chi-squared test. Correlation analysis was performed using the Spearman rho correlation coefficient. Survival analysis was performed using Kaplan-Meier curves, and the survival of different groups was compared using the log-rank test. Two-sided P-values were reported. Statistical analysis was performed using R version 3.3.3 (The R Foundation for Statistical Computing, Vienna, Austria).

## 3 Results

### 3.1 Clinical features and PD-L1 expression

**Figure 1:**
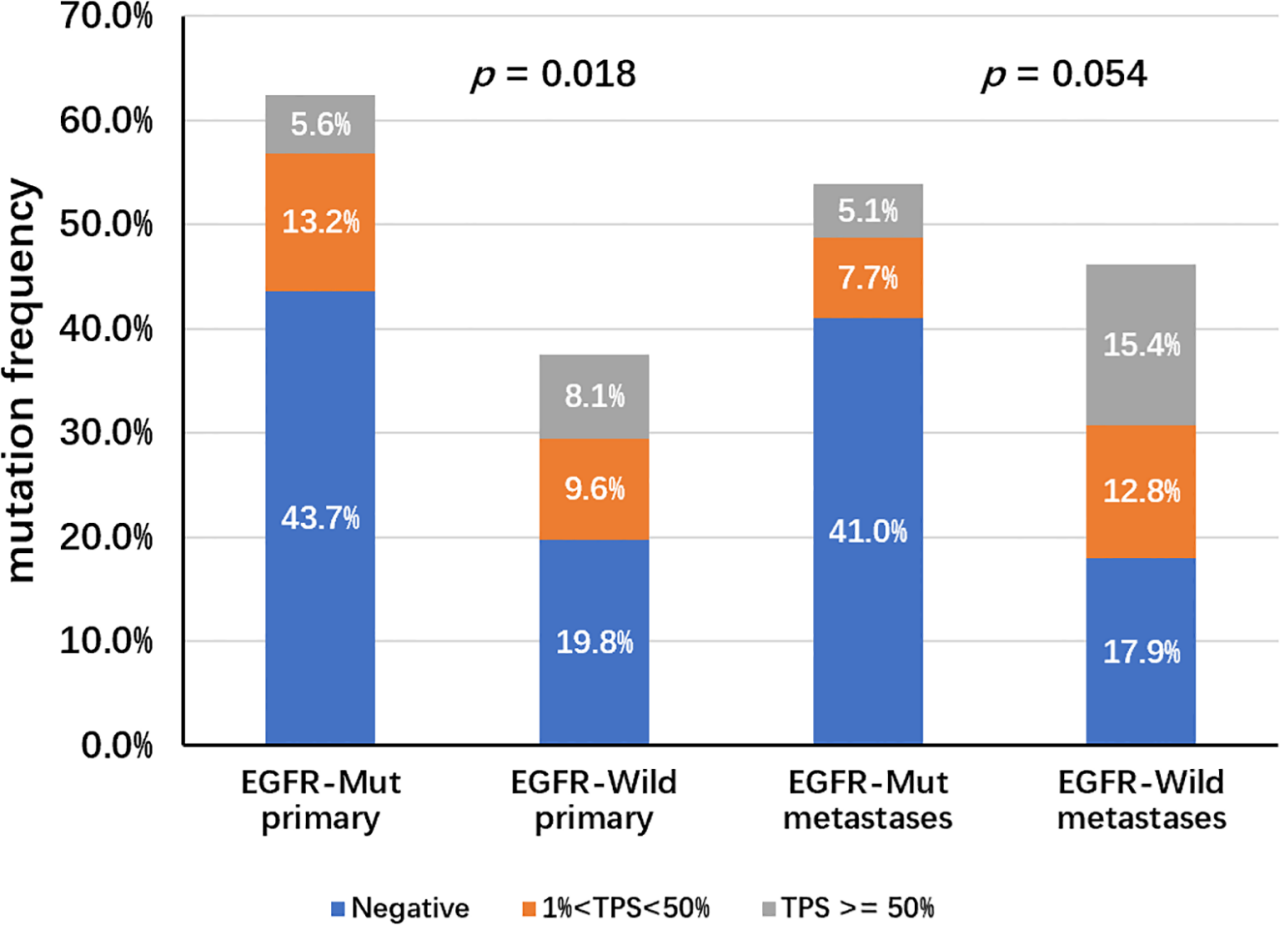
Distribution of PD-L1 expression in primary or metastatic foci, in EGFR mutation or wild-type background. In primary foci, EGFR mutant samples expressed lower levels of PD-L1 compared to wild-type samples, but there was no significant difference in metastases.

A total of 235 samples from 233 patients and corresponding information on mutations and PD-L1 expression levels were included. Two patients provided two samples, including one patient with two primary foci with different EGFR mutations, and one patient with concurrent primary and metastatic lesions. Overall, 36% (84/235) of the samples exhibited PD-L1 expression in tumor cells, including high PD-L1 expression (PD-L1 TPS ≥ 50%) in 12.4% of samples and moderate expression (1% ≤ PD-L1 TPS < 49%) in 23.3% of samples (**Table 1**). Stratification based on PD-L1 expression did not differ significantly according to the age of the patient (**Table 1**). However, higher PD-L1 expression was more common in samples from male patients (18%) compared with samples from female patients (8%). Furthermore, patients in advanced and metastatic stages (stages III and IV) exhibited significantly higher PD-L1 expression levels than those in early stages (stages I and II) (P = 0.001, **Table 1**). The proportion of PD-L1 positive expression in stage III patients was notably higher (20.4%) than in patients with stage I (2.9%) and stage II (17.4%) disease. In primary foci, samples with EGFR mutations had a higher proportion of negative PD-L1 expression (43.7% vs. 19.8%) and a lower proportion of high PD-L1 expression (5.6% vs. 8.1%) than wild-type EGFR samples (P = 0.018). However, these differences were not significant for metastatic lesions (P = 0.054; **Figure 1**).

**Table 1:** clinical characteristics and PD-L1 expression statistics of patients.

PD-L1 expression	High (N = 29)	Intermediate (N = 54)	Negative (N = 150)	P-value
Total 233 patients, 235 lesions	No. (12.4%)	No. (23.2%)	No. (64.4%)	0.13
Age (median, range)	62 (26-76)	57 (32-79)	60 (24-81)	
Gender				
Female	10	29	90	
Male	19	25	60	0.039
Stage				
I (n=70)	2	11	57	
II (n=23)	4	9	10	
III (n=49)	10	15	24	
IV (n=91)	13	19	59	0.001

### 3.2 Comparison of high-frequency mutations

**Figure 2:**
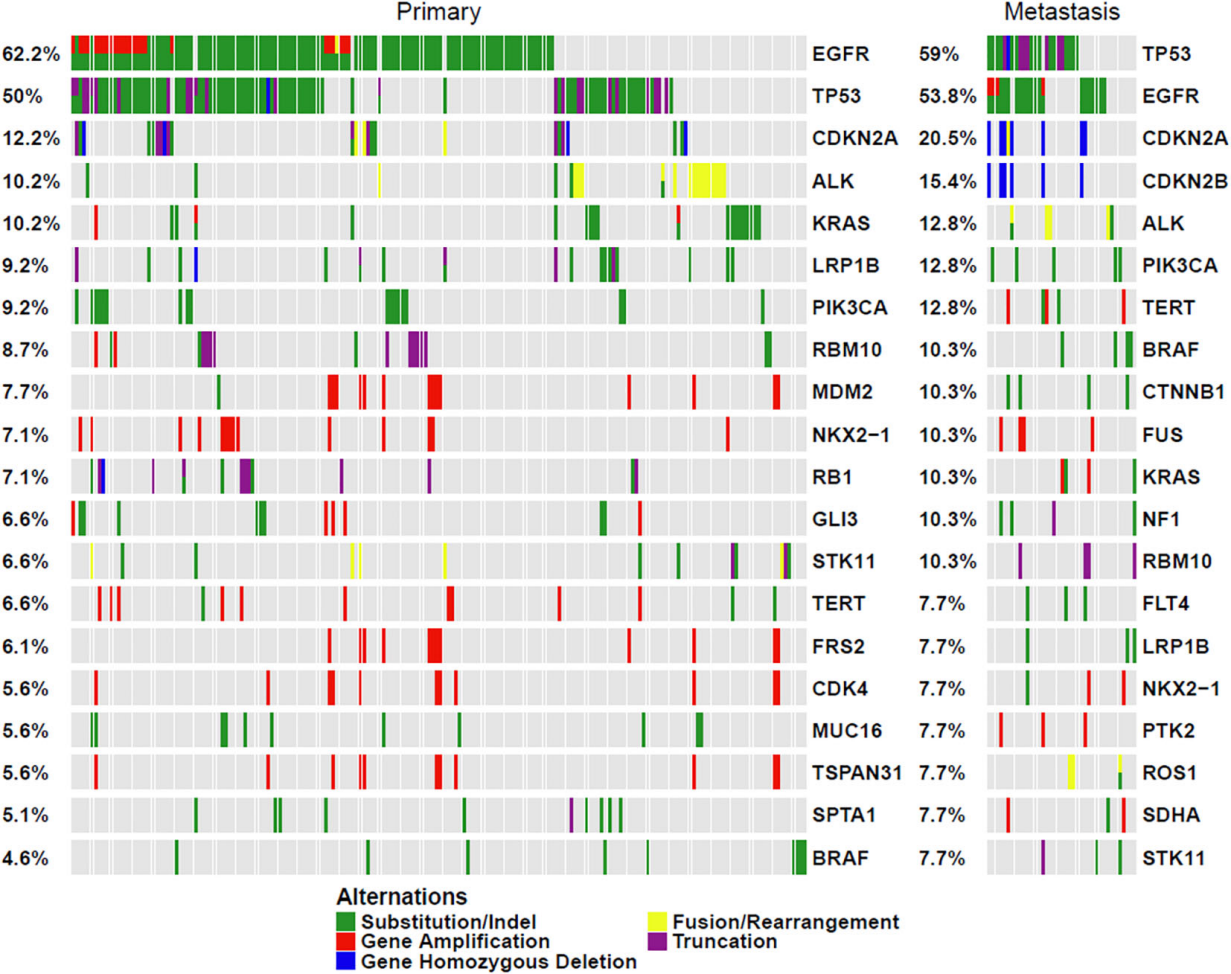
Profiling of Primary and Metastatic Lesions.

In the primary lesions in this cohort, the five most frequent mutations were in *EGFR* (62.2%), *TP53* (50%), *CDKN2A* (12.2%), *ALK* (10.2%), and *KRAS* (10.2%). Conversely, in metastatic lesions, the five most frequent mutations were in *TP53* (59%), *EGFR* (53.8%), *CDKN2A* (20.5%), *CDKN2B* (15.4%), and *ALK* (12.8%). Notably, except for the *CDKN2B* mutation, which was significantly more prevalent in metastatic lesions (P < 0.001), the other mutation frequencies did not differ significantly between primary and metastatic lesions (**Figure 2**, **Table 2**).

**Table 2:** Comparison of mutation frequency differences of high-frequency mutation genes.

Gene	Mutation in Primary	Mutation in Metastasis	Wild-type in Primary	Wild-type in Metastasis	P value
*EGFR*	122	21	74	18	0.42
*TP53*	98	23	98	16	0.40
*CDKN2A*	24	8	172	31	0.26
*ALK*	20	5	176	34	0.84
*KRAS*	20	4	176	35	1
*CDKN2B*	4	6	192	33	<0.001

### 3.3 Variations in carcinogenic pathways

**Figure 3:**
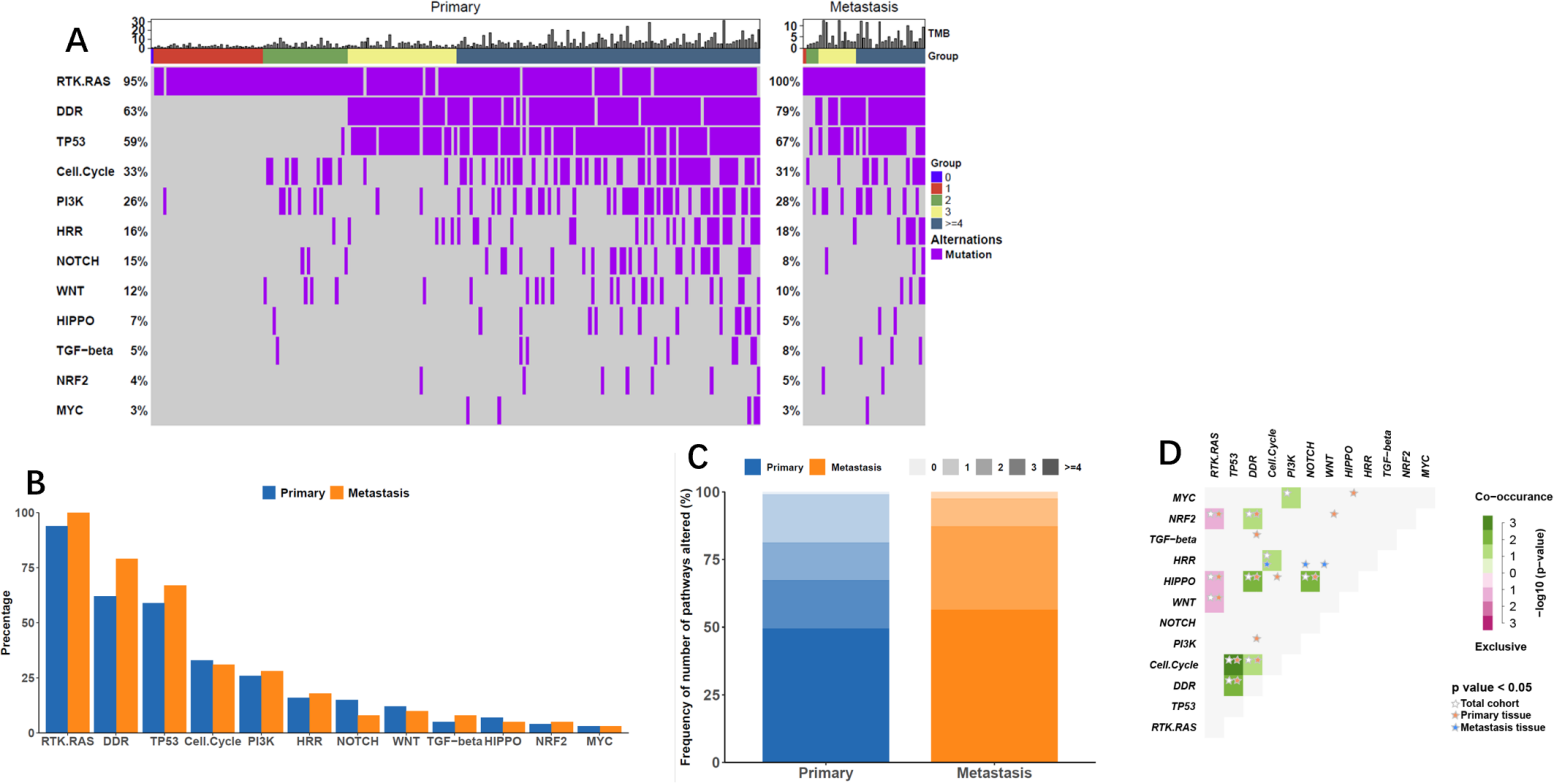
Alterations in Carcinogenic Pathways. **(A)** Oncoprint displaying changes in oncogenic pathways based on lesion subtype, showcasing the progression of pathway alterations by the number of pathways affected (NPA). **(B)** Frequency plot illustrating oncogenic pathway alterations concerning lesion subtypes. **(C)** Frequency of NPA across lesion subtypes, darker tones indicating higher alteration frequencies. **(D)** Co-occurrence and mutual exclusivity between oncogenic pathways, presented for all tumors and by lesion subtype.

Alterations in 12 gene pathways were compared in primary and metastatic tumors (**Figure 3**, **Table 3**) revealed distinct variations. Differences in specific carcinogenic pathways between primary and metastatic tumors are shown in **Figures 3A, B**. Notably, the DNA damage repair (DDR) pathway exhibited significantly greater mutation frequency in metastatic lesions (79% vs. 63%, P = 0.021). Compared with primary lesions, metastatic lesions displayed a higher proportion of altered carcinogenic pathways (**Figure 3C**, **Table 3**), particularly with NPA = 3 (30.8%) and NPA ≥ 4 (56.1%). Co-occurrence and mutual exclusivity analyses revealed frequent associations of metastatic lesions with the HRR pathway combined with the cell cycle pathway or mixed with the Notch or Wnt pathways (**Figure 3D**).

**Table 3:** Number of pathways altered (NPA) of different subgroups.

Subgroup	NPA (number of pathways altered)				
	0	1	2	3	>=4
Primary	1%	17.8%	13.8%	17.9%	49.5%
Metastasis	0	2.6%	10.3%	30.8%	56.4%

### 3.4 Association between lesion subgroup and tumor mutational burden

Although the median tumor mutational burden (TMB) of the original lesion subgroup was slightly greater than that of the metastatic lesion (4.5 mutations/Mb vs. 3.3 mutations/Mb), (P = 0.429).this difference was not statistically significant.

### 3.5 Comparison of variation types

**Figure 4:**
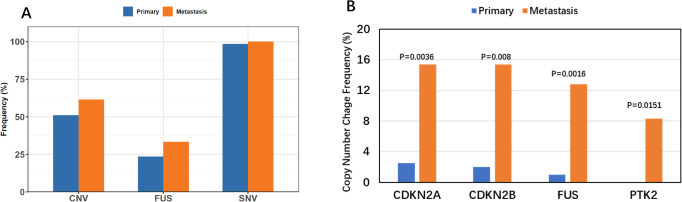
Comparison of Variation Types. **(A)** Comparison of multiple variation types across lesion subtypes, highlighting the increased frequency of CNV and FUSION in metastatic lesions. **(B)** Multiple variation types vs. lesion subtypes, showcasing significant high-frequency copy number variations in metastatic lesions for CDKN2A, CDKN2B, FUS, and PRK2 genes.

Although the number of tumors with SNVs was similar in primary and metastatic lesions (98.5% vs. 100%, P > 0.99), significant differences were noted in the prevalences of CNVs (51% vs. 61.5%, P = 0.304) and fusion (23.5% vs. 33.3%, P = 0.27); however, these differences were not statistically significant (**Figure 4A**, **Table 4**). Specifically, significant differences were found in CNVs of four genes: *CDKN2A* (homozygous deletion, 2.5% vs. 15.4%, P = 0.004*), CDKN2B* (homozygous deletion, 2% vs. 15.4%, P = 0.008), *FUS* (amplification, 1% vs. 12.8%, P = 0.002), and *PTK2* (amplification, 0.5% vs. 8.3%, P = 0.015) (**Figure 4B**, **Table 5**).

**Table 4:** Comparison of variation types of different subgroups.

subgroup	type	Variant number	Sum number	Freq	p-value
Primary	CNV	100	196	51	0.304
Metastasis	CNV	24	39	61.5	0.304
Primary	SNV	193	196	98.5	1
Metastasis	SNV	39	39	100	1
Primary	FUS	46	196	23.5	0.27
Metastasis	FUS	13	39	33.3	0.27

**Table 5:** Key CNV genes of primary and metastatic subgroups.

	*CDKN2A*	*CDKN2B*	*FUS*	*PTK2*
Primary	2.5%	2%	1%	0.5%
Metastasis	15.4%	15.4%	12.8%	8.3%
P-value	0.0036	0.008	0.0016	0.0151

### 3.6 Public data analysis of lung cancer

Previous research has shown that metastatic lung cancer frequently involves the homozygous deletion of *CDKN2A*. Using The Cancer Proteome Atlas (TCPA) database, decreased CDKN2A protein levels in lung adenocarcinoma were significantly associated with survival survival (log-rank P = 0.036, not shown). To further analyze the effects of *CDKN2A*, homozygous deletion changes in lung cancer cells were analyzed. This study examined lung cancer studies at the Memorial Sloan Kettering Cancer Center (MSKCC) using TCGA database (5, 7–13); thoracic PDX (MSK, Professional; https://www.cbioportal.org/study?id=lung_msk_pdx); and Lung Adenocarcinoma (MSKCC, Science 2015; https://www.cbioportal.org/study?id=luad_mskcc_2015).

**Figure 5:**
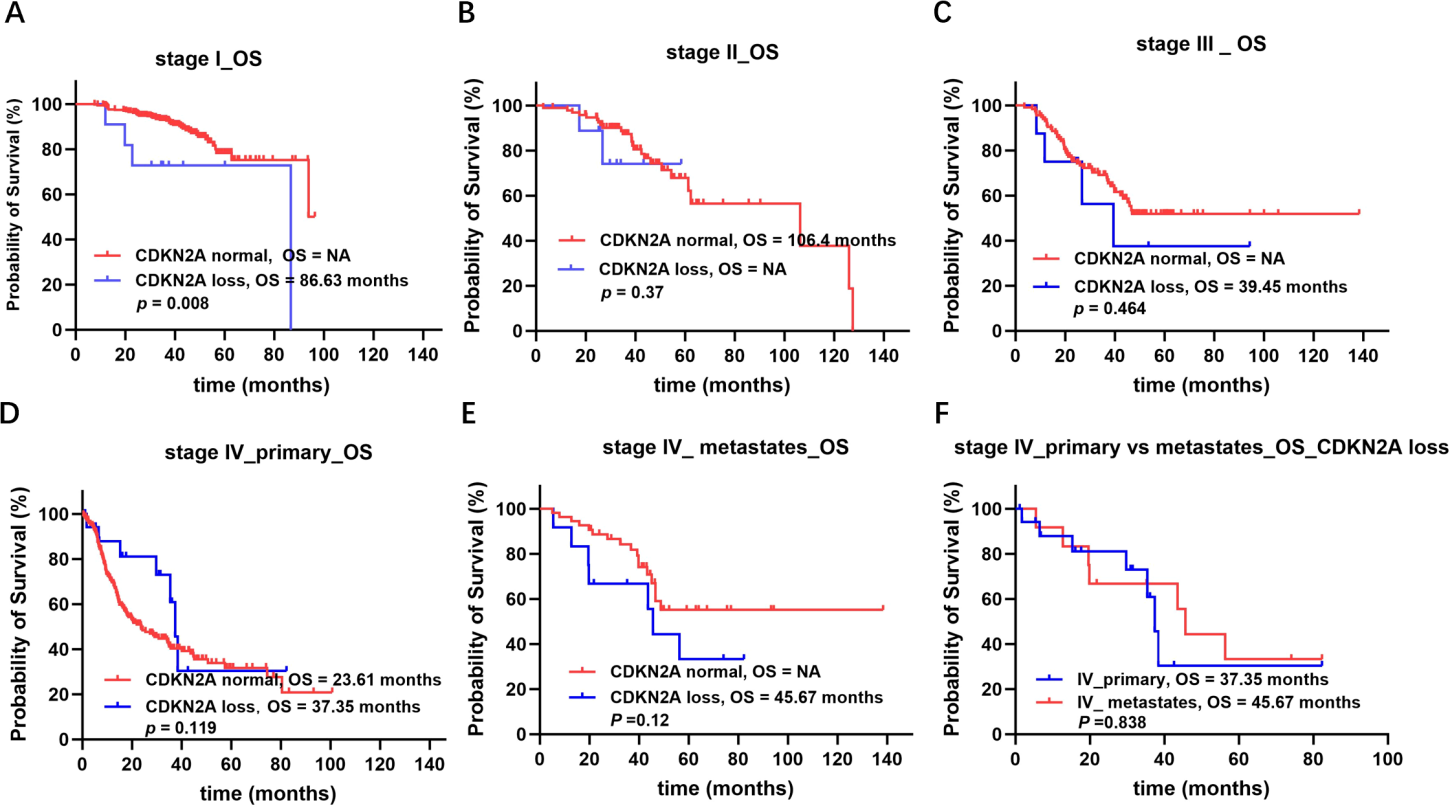
Survival analysis of lung cancer based on different stage and CDKN2A expression. **(A)** Survival analysis of stage I lung adenocarcinoma based on CDKN2A expression. **(B)** Survival analysis of stage II lung adenocarcinoma based on CDKN2A expression. **(C)** Survival analysis of stage III lung adenocarcinoma based on CDKN2A expression. **(D)** Survival analysis of stage IV lung adenocarcinoma based on CDKN2A expression of the primary tumor. **(E)** Survival analysis of stage IV lung adenocarcinoma metastases based on CDKN2A expression. **(F)** Survival analysis of stage IV lung adenocarcinoma based on the site of CDKN2A deletions.

Data were analyzed on 2532 cases of lung adenocarcinoma with information on CDKN2A expression, staging, and survival. Survival analysis revealed that in patients with stage I lung adenocarcinoma, those who lacked CDKN2A expression had worse overall survival compared with those with normal CDKN2A expression (median 86.63 months vs. not reached, P = 0.008; **Figure 5A**). However, this difference was not statistically significant in patients with stage II (median not reached vs. 106.4 months, P = 0.37; **Figure 5B**) or stage III (median not reached vs. 39.45 months, P = 0.464; **Figure 5C**) lung adenocarcinoma. In patients with stage IV lung adenocarcinoma, those who lacked CDKN2A expression in the primary tumor had longer overall survival than patients with normal CDKN2A expression (median 37.35 months vs. 23.61 months), but the difference was not significant (P = 0.119; **Figure 5D**). In patients with stage IV lung adenocarcinoma metastases, those with CDKN2A deletions had a poor prognosis (median 45.67 months vs. not reached), but the difference was not significant (P = 0.12; **Figure 5E**). Lastly, in patients with stage IV lung adenocarcinoma, those with CDKN2A deletions in the primary focus had shorter overall survival than those with CDKN2A deletions in the metastatic focus (median 37.35 months vs. 45.67), but the difference was not statistically significant (P = 0.838; **Figure 5F**).

### 3.7 Tumor sampling characteristics and PD-L1 expression

**Figure 6:**
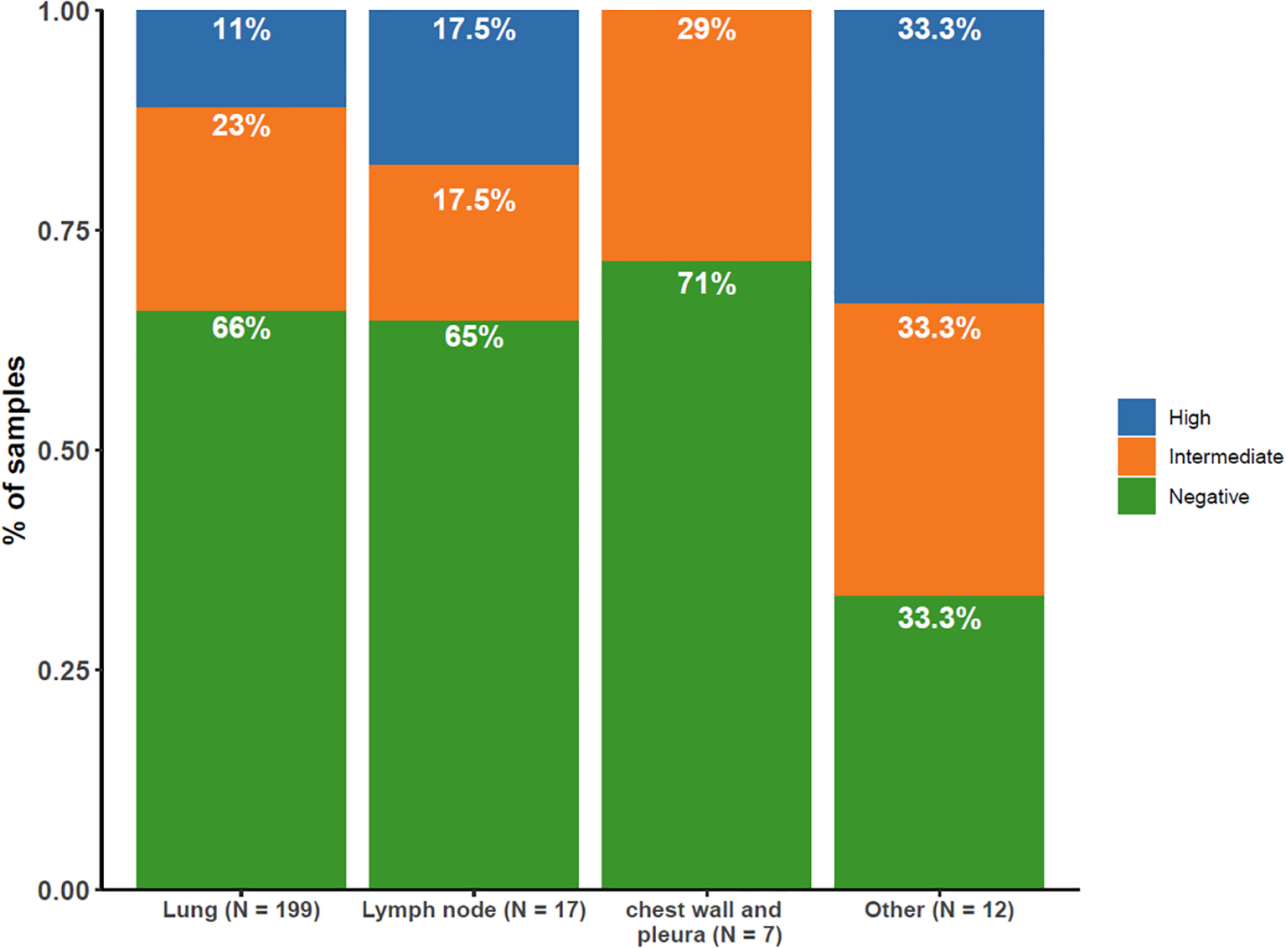
Distribution of PD-L1 Expression by Tissue Sampling Site. Illustrating the distribution of PD-L1 expression levels (high [≥50%], intermediate [1–49%], negative [<1%]) across different tissue sampling sites.

The distribution of PD-L1 expression varied according to the anatomical location (**Figure 6**). Samples from the chest wall and pleura showed no notable increase in PD-L1 expression compared with lung samples. Samples from lymph nodes were more likely to have high PD-L1 expression (17.5% vs. 11%). Notably, the liver, brain, bone, and other metastatic locations displayed the highest percentage (33.3%) of PD-L1 positive expression in this study.

### 3.8 Correlation between tumor mutational burden and PD-L1 expression

**Figure 7:**
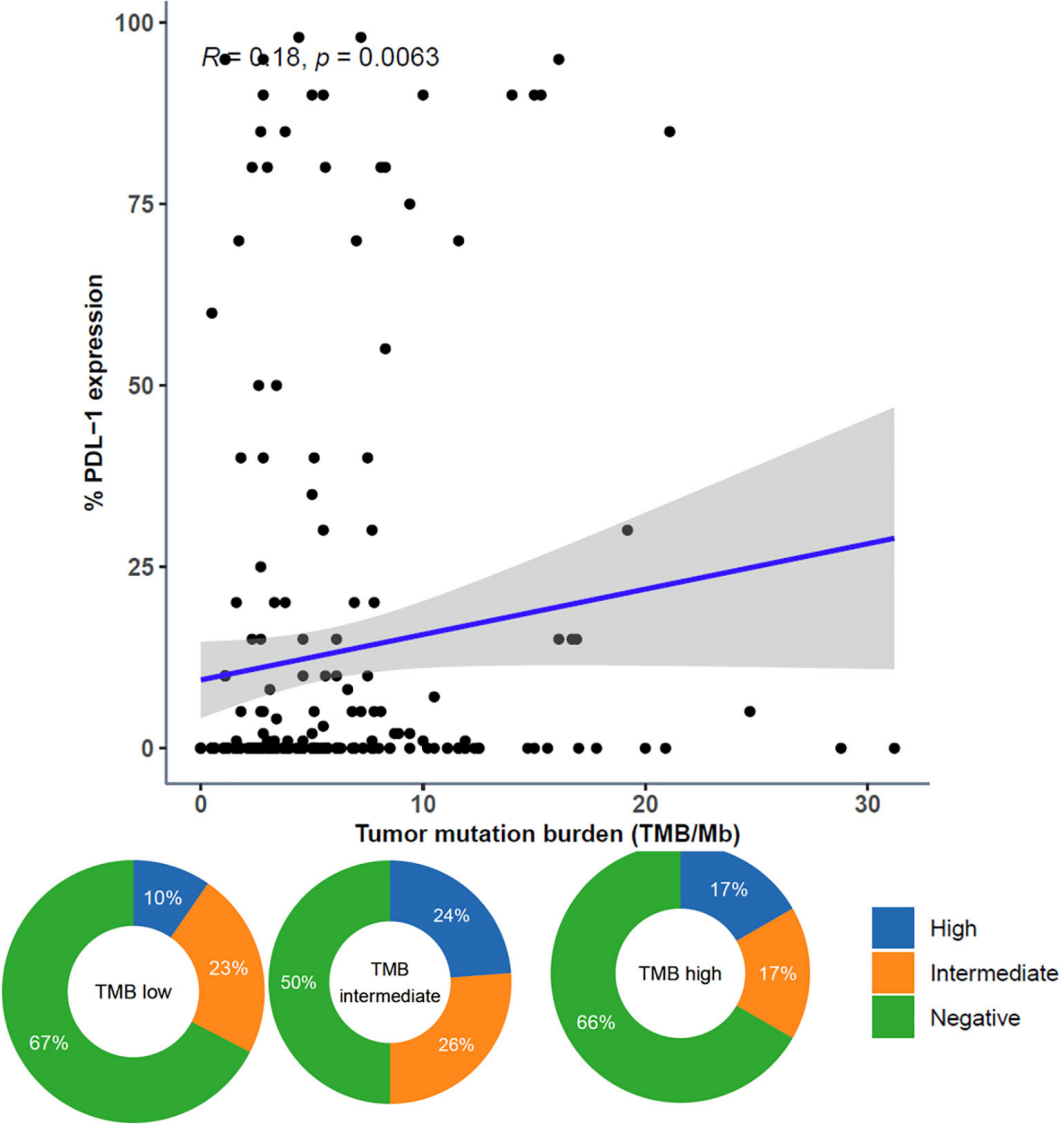
Comparison of Tumor Mutational Burden (TMB) and PD-L1 Expression. Comparison of TMB and PD-L1 expression represented as both continuous variables (above; dots representing individual tumor samples; Spearman rho=0.18, p=0.0063) and categorical variables (below; donut plots showing the proportion of patients with PD-L1 high [green] within TMB subgroups [TMB low < 10 muts/mb, TMB intermediate ≥ 10 muts/mb and < 20 muts/mb, TMB high ≥ 20 muts/mb]).

The continuous variables TMB and PD-L1 expression were moderately correlated. When categorized, PD-L1 high was more prevalent in TMB intermediate and TMB high samples than in TMB low samples (**Figure 7**).

## 4 Discussion

Highly prevalent mutations in genes such as *EGFR*, *KRAS*, and *ALK* contribute substantially to lung adenocarcinoma and are pivotal for recommending targeted therapies (14, 15). PD-L1 expression is a crucial marker for lung cancer immunotherapy (16, 17). Notably, although *KRAS* mutations often indicate a heightened response to immunotherapy, this advantage is commonly counterbalanced by additional *TP53* mutations (18). Notably, patients with wild-type KRAS and TP53 who received adjuvant chemotherapy at any stage demonstrated prolonged overall survival (OS) compared with those with single or double *TP53* or *KRAS* mutations (19). These intricate mutation factors profoundly affect survival, necessitating personalized selection of therapy to optimize prognosis. In this extensive study, we performed a comparison of gene mutations and PD-L1 expression levels in samples from patients with primary and metastatic lung adenocarcinoma treated at Shandong Provincial Hospital in China.

Currently, PD-L1-based immunotherapy has evolved from being the primary treatment for metastatic disease to being used as a neoadjuvant therapy for patients with early-stage disease (20). Notably, among patients with stage III disease in our cohort, a higher proportion exhibited elevated PD-L1 expression, suggesting the potential for adopting neoadjuvant immunotherapy strategies to achieve tumor regression and enable subsequent surgical eradication. Moreover, leveraging genetic mutation data from the primary lesion can mitigate risk factors and augment the effectiveness of neoadjuvant immunotherapy. These patients might benefit from CDK4 inhibitors because of lowered CDKN2A/CDKN2B levels, which enhance CDK4/cyclin D activity.

In this cohort, metastatic lesions exhibited a higher prevalence of changes within the carcinogenic pathways compared with primary lesions. Specifically, the frequency of mutations in the DDR pathway within the metastatic lesions was notably elevated. Previous studies have shown that increased alterations in the DDR pathway augment the effectiveness of immunotherapy, often resulting in increased TMB and extended OS (21). Consequently, concurrent alteration of the DDR pathway, along with a higher PD-L1 expression rate, holds promise for enhancing the effectiveness of immunotherapy in patients with metastatic lesions. Moreover, in metastatic lesions, the HRR gene pathway frequently occurs with the cell cycle pathway or with alterations in the Notch or Wnt pathways. This suggests that metastatic sites tend to rely more on changes in the DDR or HRR pathways to induce genomic instability. Notably, metastatic lung cancer lesions have a higher degree of genomic instability than the primary lung lesion, consistent with the findings of a previous study (3).

Previous research has highlighted that lung cancer metastasis is not strongly linked to SNV, but is more strongly associated with chromosome instability (3). This instability results from a deficiency in DNA repair enzymes, often leading to chromosome breakage, deletion, or rearrangement (22). In our study, we found a significantly higher incidence of CNV and fusion in metastatic lesions, in contrast with the lower CNV levels observed in primary lesions. Lower CNV levels have previously been shown to be associated with extended progression-free survival in patients with lung adenocarcinoma undergoing radiotherapy (23). Similarly, among patients with advanced non-small cell lung cancer (NSCLC) treated with anti-PD-L1 therapy, those with sustained clinical benefits have lower CNV levels than those without persistent benefits (24). Hence, patients with primary lung cancer with low CNV levels may benefit from chemotherapy and immunotherapy.

This study revealed a substantial increase in the number of copy number deletions of *CDKN2A* and *CDKN2B* in metastatic lesions. These genes are located adjacently on chromosome 9. Specifically, compared with primary tumors, metastatic lung cancer displayed a higher absence of CDKN2A. CDKN2A inactivation is common in lung cancer, caused by homozygous deletion, promoter region methylation, or point mutations (25). The homozygous deletion form of CDKN2A is common in patients with lung adenocarcinoma, with a prevalence of 22% in Chinese population; TCGA data of people of European descent shows a mutation frequency of 15%; whereas Korean data shows only a 4% prevalence of *CDKN2A* mutations (26). In patients with stage I–III lung cancer, CDKN2A deletion has been linked to poorer disease-free survival (27). Additionally, a significant loss of CDKN2A copy number, ranging from 27% to 28%, has been observed in patients with lung cancer with brain metastasis (4, 28).

The lung cancer data retrieved from the TCPA database showed that lower expression levels of p16/CDKN2A protein was associated with poorer OS. Additionally, insights gleaned from MSK data within the TCGA database revealed that in patients with stage I lung cancer, the loss of CDKN2A copy numbers was associated with poor OS. Genomic changes in *CDKN2A* in early NSCLC are associated with recurrence. Previous studies have also shown that the co-occurrence of *p16*/*CDKN2A* homozygous deletion and activated *EGFR* mutation in patients with lung adenocarcinoma results in decreased responsiveness to EGFR-TKIs (29, 30). Hence, it is important to diagnose and treat lung cancer early in individuals with homozygous deletion of *CDKN2A*.

This study showed a moderate positive correlation between TMB and the level of PD-L1 expression. Previous studies have shown that higher TMB levels are correlated with increased immune cell infiltration and a robust T-cell-mediated inflammatory response, thereby enhancing the sensitivity of the PD-L1 expression subgroup to PD-L1/PD-1 immune blockade. Consequently, for patients with metastases, positive PD-L1 expression coupled with higher TMB levels suggests a potentially more favorable response to PD-L1 blocking immunotherapy.

This study revealed elevated PD-L1 expression in patients with lymph node metastases, which is consistent with the findings of previous studies and has been shown to be associated with poorer prognosis (5). Consequently, increased attention to the clinical follow-up of patients with lymph node metastases is warranted, and further evaluation of the effect of targeted treatment in patients with lymph node metastases is required.

This study contributes to documenting the differences in gene mutations between primary and metastatic lung adenocarcinoma in Shandong Province, China. Building on this foundation, we propose enhancing the clinical management and post-treatment monitoring of patients with *CDKN2A* homozygous deletions, particularly those with metastases. However, our findings are constrained by the limited sample size. Further studies of larger clinical cohorts are needed to provide a more comprehensive analysis, deepening our understanding of molecular prognostic markers in patients with lung cancer with distant metastases. The findings of this study are consistent with those of previous studies on NSCLC, and provide additional insights.

## 5 Conclusion

This study investigated the PD-L1 expression levels and gene variants in patients with primary and metastatic lung adenocarcinoma in Shandong Province, China. These results provide valuable insights into real-world clinical data on lung cancer.

## Data Availability

The raw data supporting the conclusions of this article will be made available by the authors, without undue reservation.
